# Crystal structure of 4-methyl-*N*-[(4-methyl­pyridin-2-yl)carbamo­thioyl]­benzamide

**DOI:** 10.1107/S2056989015003412

**Published:** 2015-02-28

**Authors:** Farook Adam, Nadiah Ameram, Naser Eltaher Eltayeb

**Affiliations:** a School of Chemical Sciences, 11800, USM Pulau Pinang, Malaysia; b College of Sciences and Arts, Rabigh, King Abdulaziz University, Saudi Arabia, and International University of Africa, Khartoum, Sudan

**Keywords:** crystal structure, thio­urea compounds, thio­carbonyl groups, benzamide, hydrogen bonding

## Abstract

In the title compound, C_15_H_15_N_3_OS, the dihedral angle between the planes of the benzene and pyridine rings is 26.86 (9)°. Intra­molecular N—H⋯O and C—H⋯S hydrogen bonds both generate *S*(6) rings. The C=O and C=S bonds lie to opposite sides of the mol­ecule. In the crystal, inversion dimers linked by pairs of N—H⋯S hydrogen bonds generate *R*_2_^2^(8) loops.

## Abstract 2

In the title compound, C_15_H_15_N_3_OS, intra­molecular N—H⋯O and C—H⋯S hydrogen bonds both generate *S*(6) rings. The C=O and C=S bonds lie to opposite sides of the mol­ecule. In the crystal, inversion dimers linked by pairs of N—H⋯S hydrogen bonds generate 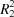
(8) loops.

## Chemical context   

The role of benzoyl thio­urea derivatives in coordination chemistry has been extensively studied and quite satisfactorily elucidated. As benzoyl thio­ureas have suitable C=O and C=S functional groups, they can be considered as useful chelating agents due to their ability to encapsulate metal ions into their coordinating moiety. Thio­urea and its derivatives have found extensive applications in the fields of medicine, agriculture and analytical chemistry. Thio­ureas are also known to exhibit a wide range of biological activities including anti­cancer (Saeed *et al.*, 2010*a* ▸), anti­fungal (Saeed *et al.*, 2010*b* ▸) and as agrochemicals (Xu *et al.*, 2003 ▸). As part of our studies in this area, we now describe the synthesis and structure of the title compound, (I).
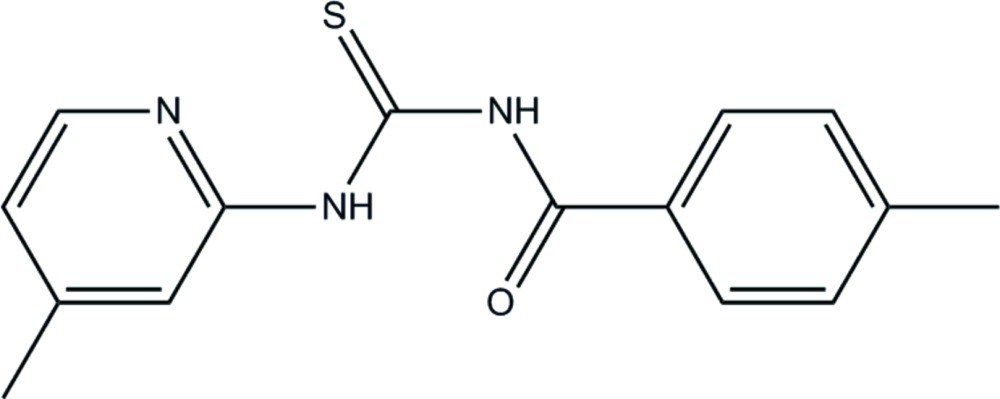


## Structural commentary   

**Figure 1:**
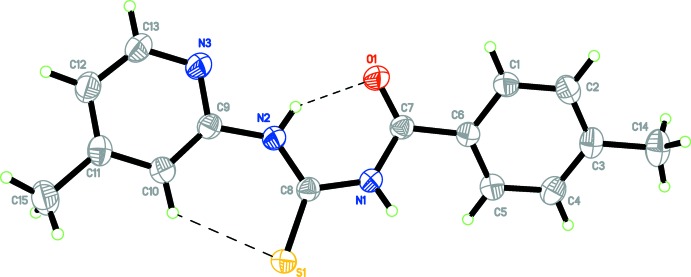
The mol­ecular structure of the title compound, with 50% probability displacement ellipsoids. Hydrogen bonds are shown as dashed lines.

The title compound (Fig. 1 ▸) is a benzoyl thio­urea derivative and analogous to a compound recently reported by us (Adam *et al.*, 2014 ▸), except that the other substituent is changed to methyl­pyridine and the thio­urea moiety is still in a *para* position. The dihedral angle between the planes of the benzene and pyridine rings is 26.86 (9)°. The C=O bond length of 1.225 (2) Å is comparable to that observed in *N*-benzoyl-*N*′-phenyl­thio­urea (Hassan *et al.*, 2008*a* ▸). The C—N bond lengths are in the range 1.328 (2)–1.417 (2) Å, shorter than the normal single C—N bond length (1.469 Å), indicating partial double-bond character owing to the resonance effect at the carbonyl­thio­urea moiety.

As in most benzoyl thio­urea derivatives, an intra­molecular N—H⋯O hydrogen bond leads to the formation of an *S*(6) ring, namely, C7/N1/C8/N2/H2/O1. An intra­molecular C—H⋯S inter­action (C9/N2/C8/S1/H10/C10) also generates an *S*(6) ring (Fig. 1 ▸, Table 1 ▸).

## Supra­molecular features   

**Figure 2:**
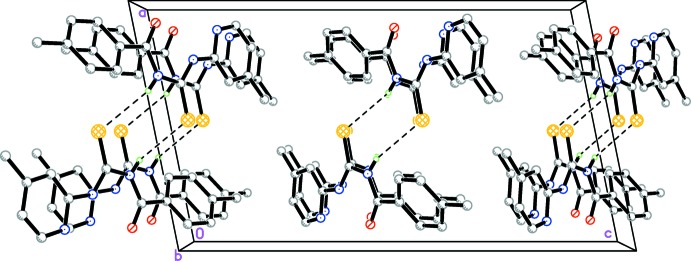
The crystal packing of the title compound viewed down the *c* axis. Hydrogen bonds are shown as dashed lines.

In the crystal of (I), inversion dimers linked by pairs of N—H⋯S hydrogen bonds (Table 1 ▸, Fig. 2 ▸) generate 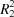
(8) loops. As free rotation about the N1—C7 and N2—C8 single bonds is hindered, the C=O and C=S bonds are unlikely to align at the same side of the mol­ecule in order to form a chelate with a metal ion.

## Synthesis and crystallization   

The title compound was prepared according to a slight modification of the method described by Hassan *et al.* (2008*b* ▸). *p*-Benzoyl chloride (13 mmol) was added dropwise to a stirred acetone solution (30 ml) of ammonium thio­cyanate (13 mmol). The mixture was stirred for 10 min. A solution of 2-amino-4-picoline in acetone was added and the reaction mixture was refluxed for 3 h, after which the solution was poured into a beaker containing some ice cubes. The resulting precipitate was collected by filtration, washed several times with a cold ethanol/water mixture and purified by recrystallization from an ethanol solution.

## Refinement   

Crystal data, data collection and structure refinement details are summarized in Table 2 ▸. The H-atoms on the N atoms were located in a difference-Fourier map and were freely refined. All other H atoms were positioned geometrically and refined using a riding model with C—H = 0.93–0.96 Å and *U*_iso_(H) = 1.2*U*_eq_(aromatic C) or 1.5*U*_eq_(methyl C).

## Supplementary Material

Crystal structure: contains datablock(s) I. DOI: 10.1107/S2056989015003412/hb7367sup1.cif

Structure factors: contains datablock(s) I. DOI: 10.1107/S2056989015003412/hb7367Isup2.hkl

Supporting information file. DOI: 10.1107/S2056989015003412/hb7367Isup3.cml

CCDC reference: 1050132

Additional supporting information:  crystallographic information; 3D view; checkCIF report

## Figures and Tables

**Table 1:** Hydrogen-bond geometry (, )

*D*H*A*	*D*H	H*A*	*D* *A*	*D*H*A*
N2H1*N*2O1	0.82(2)	1.94(2)	2.644(2)	144(2)
N1H1*N*1S1^i^	0.81(2)	2.74(2)	3.511(2)	158(2)
C10H10*A*S1	0.93	2.57	3.221(2)	127

Symmetry code: (i) 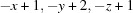.

**Table 2:** Experimental details

Crystal data	
Chemical formula	C_15_H_15_N_3_OS
*M*_r_	285.36
Crystal system, space group	Monoclinic, *P*2_1_/*c*
Temperature (K)	294
*a*, *b*, *c* ()	11.5297(12), 6.1860(6), 20.657(2)
()	101.431(2)
*V* (^3^)	1444.1(3)
*Z*	4
Radiation type	Mo *K*
(mm^1^)	0.22
Crystal size (mm)	0.38 0.34 0.09

Data collection	
Diffractometer	Bruker APEXII CCD
Absorption correction	Multi-scan (*SADABS*; Bruker, 2005 ▸)
*T*_min_, *T*_max_	0.920, 0.981
No. of measured, independent and observed [*I* > 2(*I*)] reflections	15813, 4233, 2790
*R*_int_	0.028
(sin /)_max_ (^1^)	0.706

Refinement	
*R*[*F* ^2^ > 2(*F* ^2^)], *wR*(*F* ^2^), *S*	0.048, 0.164, 1.05
No. of reflections	4233
No. of parameters	191
H-atom treatment	H atoms treated by a mixture of independent and constrained refinement
_max_, _min_ (e ^3^)	0.27, 0.19

Computer programs: *APEX2* and *SAINT* (Bruker, 2005 ▸), *SHELXTL* (Sheldrick, 2008 ▸) and *PLATON* (Spek, 2009 ▸).

## supplementary crystallographic information

### Crystal data

**Table:**

C_15_H_15_N_3_OS	*F*(000) = 600
*M**_r_* = 285.36	*D*_x_ = 1.313 Mg m^−^^3^
Monoclinic, *P*2_1_/*c*	Mo *K*α radiation, λ = 0.71073 Å
Hall symbol: -P 2ybc	Cell parameters from 3960 reflections
*a* = 11.5297 (12) Å	θ = 2.4–25.9°
*b* = 6.1860 (6) Å	µ = 0.22 mm^−^^1^
*c* = 20.657 (2) Å	*T* = 294 K
β = 101.431 (2)°	Plate, colourless
*V* = 1444.1 (3) Å^3^	0.38 × 0.34 × 0.09 mm
*Z* = 4	

### Data collection

**Table:**

Bruker APEXII CCD diffractometer	4233 independent reflections
Radiation source: fine-focus sealed tube	2790 reflections with *I* > 2σ(*I*)
Graphite monochromator	*R*_int_ = 0.028
φ and ω scans	θ_max_ = 30.1°, θ_min_ = 1.8°
Absorption correction: multi-scan (*SADABS*; Bruker, 2005)	*h* = −16→16
*T*_min_ = 0.920, *T*_max_ = 0.981	*k* = −8→8
15813 measured reflections	*l* = −29→29

### Refinement

**Table:**

Refinement on *F*^2^	Primary atom site location: structure-invariant direct methods
Least-squares matrix: full	Secondary atom site location: difference Fourier map
*R*[*F*^2^ > 2σ(*F*^2^)] = 0.048	Hydrogen site location: inferred from neighbouring sites
*wR*(*F*^2^) = 0.164	H atoms treated by a mixture of independent and constrained refinement
*S* = 1.05	*w* = 1/[σ^2^(*F*_o_^2^) + (0.0866*P*)^2^ + 0.1249*P*] where *P* = (*F*_o_^2^ + 2*F*_c_^2^)/3
4233 reflections	(Δ/σ)_max_ < 0.001
191 parameters	Δρ_max_ = 0.27 e Å^−^^3^
0 restraints	Δρ_min_ = −0.19 e Å^−^^3^

### Special details

**Table:**

Geometry. All e.s.d.'s (except the e.s.d. in the dihedral angle between two l.s. planes) are estimated using the full covariance matrix. The cell e.s.d.'s are taken into account individually in the estimation of e.s.d.'s in distances, angles and torsion angles; correlations between e.s.d.'s in cell parameters are only used when they are defined by crystal symmetry. An approximate (isotropic) treatment of cell e.s.d.'s is used for estimating e.s.d.'s involving l.s. planes.
Refinement. Refinement of *F*^2^ against ALL reflections. The weighted *R*-factor *wR* and goodness of fit *S* are based on *F*^2^, conventional *R*-factors *R* are based on *F*, with *F* set to zero for negative *F*^2^. The threshold expression of *F*^2^ > σ(*F*^2^) is used only for calculating *R*-factors(gt) *etc*. and is not relevant to the choice of reflections for refinement. *R*-factors based on *F*^2^ are statistically about twice as large as those based on *F*, and *R*- factors based on ALL data will be even larger.

### Fractional atomic coordinates and isotropic or equivalent isotropic displacement parameters (Å^2^)

**Table:**

	*x*	*y*	*z*	*U*_iso_*/*U*_eq_	
S1	0.47752 (4)	0.82687 (9)	0.41107 (2)	0.0655 (2)	
O1	0.10758 (10)	1.0358 (2)	0.43058 (7)	0.0664 (4)	
N1	0.30931 (13)	1.0520 (2)	0.44717 (7)	0.0489 (3)	
N2	0.24183 (13)	0.7757 (3)	0.37524 (7)	0.0499 (3)	
N3	0.11827 (13)	0.5315 (3)	0.31789 (8)	0.0656 (4)	
C1	0.11290 (16)	1.3270 (3)	0.53813 (9)	0.0549 (4)	
H1A	0.0514	1.2271	0.5317	0.066*	
C2	0.11387 (17)	1.4947 (3)	0.58230 (9)	0.0618 (5)	
H2A	0.0534	1.5049	0.6060	0.074*	
C3	0.20271 (18)	1.6472 (3)	0.59200 (9)	0.0585 (5)	
C4	0.29222 (18)	1.6283 (3)	0.55643 (9)	0.0595 (5)	
H4A	0.3525	1.7306	0.5622	0.071*	
C5	0.29362 (16)	1.4598 (3)	0.51247 (8)	0.0525 (4)	
H5A	0.3547	1.4492	0.4892	0.063*	
C6	0.20408 (14)	1.3073 (3)	0.50317 (8)	0.0463 (4)	
C7	0.20039 (14)	1.1224 (3)	0.45721 (8)	0.0481 (4)	
C8	0.33584 (14)	0.8813 (3)	0.40921 (7)	0.0456 (4)	
C9	0.23136 (14)	0.5873 (3)	0.33544 (7)	0.0485 (4)	
C10	0.32251 (15)	0.4763 (3)	0.31581 (8)	0.0546 (4)	
H10A	0.4004	0.5224	0.3292	0.065*	
C11	0.29672 (18)	0.2966 (3)	0.27621 (8)	0.0549 (4)	
C12	0.18012 (19)	0.2358 (4)	0.25798 (10)	0.0673 (5)	
H12A	0.1591	0.1146	0.2316	0.081*	
C13	0.0953 (2)	0.3570 (4)	0.27931 (12)	0.0766 (6)	
H13A	0.0167	0.3150	0.2661	0.092*	
C14	0.2033 (3)	1.8322 (4)	0.64013 (11)	0.0836 (7)	
H14A	0.2708	1.9228	0.6397	0.125*	
H14B	0.2073	1.7754	0.6838	0.125*	
H14C	0.1322	1.9156	0.6274	0.125*	
C15	0.3924 (2)	0.1738 (4)	0.25264 (13)	0.0834 (7)	
H15A	0.4636	0.1785	0.2858	0.125*	
H15B	0.4068	0.2380	0.2126	0.125*	
H15C	0.3682	0.0262	0.2443	0.125*	
H1N2	0.181 (2)	0.810 (4)	0.3871 (12)	0.083 (7)*	
H1N1	0.3673 (18)	1.100 (3)	0.4720 (10)	0.055 (5)*	

### Atomic displacement parameters (Å^2^)

**Table:**

	*U*^11^	*U*^22^	*U*^33^	*U*^12^	*U*^13^	*U*^23^
S1	0.0427 (3)	0.0886 (4)	0.0651 (3)	−0.0032 (2)	0.0107 (2)	−0.0266 (2)
O1	0.0437 (7)	0.0727 (10)	0.0793 (9)	0.0008 (6)	0.0035 (6)	−0.0220 (7)
N1	0.0440 (7)	0.0525 (9)	0.0488 (7)	−0.0028 (6)	0.0057 (6)	−0.0086 (6)
N2	0.0429 (7)	0.0548 (9)	0.0498 (7)	0.0016 (6)	0.0040 (6)	−0.0088 (6)
N3	0.0508 (8)	0.0691 (11)	0.0723 (10)	−0.0025 (8)	0.0012 (7)	−0.0189 (8)
C1	0.0478 (9)	0.0604 (11)	0.0555 (9)	0.0019 (8)	0.0083 (7)	−0.0018 (8)
C2	0.0624 (11)	0.0725 (13)	0.0510 (9)	0.0149 (10)	0.0124 (8)	−0.0027 (8)
C3	0.0731 (12)	0.0516 (11)	0.0464 (9)	0.0149 (9)	0.0011 (8)	0.0002 (7)
C4	0.0701 (12)	0.0457 (10)	0.0602 (10)	−0.0014 (9)	0.0066 (9)	0.0008 (8)
C5	0.0597 (10)	0.0466 (10)	0.0520 (9)	0.0011 (8)	0.0127 (7)	0.0038 (7)
C6	0.0475 (8)	0.0447 (9)	0.0452 (8)	0.0044 (7)	0.0053 (6)	0.0016 (6)
C7	0.0454 (8)	0.0494 (10)	0.0480 (8)	0.0005 (7)	0.0054 (7)	−0.0004 (7)
C8	0.0457 (8)	0.0501 (9)	0.0401 (7)	−0.0016 (7)	0.0064 (6)	−0.0001 (6)
C9	0.0495 (9)	0.0526 (10)	0.0407 (7)	−0.0004 (7)	0.0029 (6)	−0.0006 (7)
C10	0.0548 (10)	0.0566 (11)	0.0534 (9)	−0.0008 (8)	0.0131 (7)	−0.0033 (8)
C11	0.0730 (12)	0.0469 (10)	0.0460 (8)	0.0014 (8)	0.0148 (8)	0.0007 (7)
C12	0.0770 (13)	0.0601 (12)	0.0648 (11)	−0.0072 (10)	0.0138 (10)	−0.0136 (9)
C13	0.0624 (12)	0.0760 (15)	0.0862 (14)	−0.0093 (10)	0.0022 (10)	−0.0282 (12)
C14	0.114 (2)	0.0673 (15)	0.0669 (13)	0.0168 (12)	0.0112 (13)	−0.0144 (10)
C15	0.0940 (17)	0.0733 (16)	0.0907 (16)	0.0025 (12)	0.0374 (14)	−0.0172 (12)

### Geometric parameters (Å, º)

**Table:**

S1—C8	1.6609 (16)	C4—H4A	0.9300
O1—C7	1.225 (2)	C5—C6	1.384 (2)
N1—C7	1.383 (2)	C5—H5A	0.9300
N1—C8	1.385 (2)	C6—C7	1.482 (2)
N1—H1N1	0.81 (2)	C9—C10	1.382 (2)
N2—C8	1.339 (2)	C10—C11	1.377 (3)
N2—C9	1.417 (2)	C10—H10A	0.9300
N2—H1N2	0.82 (2)	C11—C12	1.375 (3)
N3—C9	1.328 (2)	C11—C15	1.498 (3)
N3—C13	1.337 (3)	C12—C13	1.373 (3)
C1—C2	1.380 (3)	C12—H12A	0.9300
C1—C6	1.394 (2)	C13—H13A	0.9300
C1—H1A	0.9300	C14—H14A	0.9600
C2—C3	1.378 (3)	C14—H14B	0.9600
C2—H2A	0.9300	C14—H14C	0.9600
C3—C4	1.385 (3)	C15—H15A	0.9600
C3—C14	1.515 (3)	C15—H15B	0.9600
C4—C5	1.385 (3)	C15—H15C	0.9600
			
C7—N1—C8	129.34 (15)	N2—C8—S1	127.11 (13)
C7—N1—H1N1	116.6 (14)	N1—C8—S1	117.91 (12)
C8—N1—H1N1	112.9 (14)	N3—C9—C10	123.61 (16)
C8—N2—C9	132.25 (15)	N3—C9—N2	109.77 (15)
C8—N2—H1N2	111.7 (17)	C10—C9—N2	126.61 (15)
C9—N2—H1N2	114.2 (17)	C11—C10—C9	119.22 (17)
C9—N3—C13	116.14 (17)	C11—C10—H10A	120.4
C2—C1—C6	120.01 (18)	C9—C10—H10A	120.4
C2—C1—H1A	120.0	C12—C11—C10	117.90 (18)
C6—C1—H1A	120.0	C12—C11—C15	121.03 (18)
C3—C2—C1	121.29 (17)	C10—C11—C15	121.07 (19)
C3—C2—H2A	119.4	C13—C12—C11	118.87 (19)
C1—C2—H2A	119.4	C13—C12—H12A	120.6
C2—C3—C4	118.38 (17)	C11—C12—H12A	120.6
C2—C3—C14	121.3 (2)	N3—C13—C12	124.3 (2)
C4—C3—C14	120.3 (2)	N3—C13—H13A	117.9
C5—C4—C3	121.21 (18)	C12—C13—H13A	117.9
C5—C4—H4A	119.4	C3—C14—H14A	109.5
C3—C4—H4A	119.4	C3—C14—H14B	109.5
C6—C5—C4	119.94 (17)	H14A—C14—H14B	109.5
C6—C5—H5A	120.0	C3—C14—H14C	109.5
C4—C5—H5A	120.0	H14A—C14—H14C	109.5
C5—C6—C1	119.15 (16)	H14B—C14—H14C	109.5
C5—C6—C7	122.77 (15)	C11—C15—H15A	109.5
C1—C6—C7	118.08 (15)	C11—C15—H15B	109.5
O1—C7—N1	122.22 (16)	H15A—C15—H15B	109.5
O1—C7—C6	122.46 (15)	C11—C15—H15C	109.5
N1—C7—C6	115.31 (15)	H15A—C15—H15C	109.5
N2—C8—N1	114.97 (14)	H15B—C15—H15C	109.5
			
C6—C1—C2—C3	1.2 (3)	C9—N2—C8—N1	−174.80 (16)
C1—C2—C3—C4	−0.3 (3)	C9—N2—C8—S1	4.2 (3)
C1—C2—C3—C14	179.57 (18)	C7—N1—C8—N2	3.2 (3)
C2—C3—C4—C5	−0.5 (3)	C7—N1—C8—S1	−175.97 (14)
C14—C3—C4—C5	179.64 (17)	C13—N3—C9—C10	0.3 (3)
C3—C4—C5—C6	0.4 (3)	C13—N3—C9—N2	179.18 (18)
C4—C5—C6—C1	0.5 (3)	C8—N2—C9—N3	173.13 (18)
C4—C5—C6—C7	−179.68 (16)	C8—N2—C9—C10	−8.0 (3)
C2—C1—C6—C5	−1.2 (3)	N3—C9—C10—C11	−0.4 (3)
C2—C1—C6—C7	178.91 (16)	N2—C9—C10—C11	−179.18 (16)
C8—N1—C7—O1	−1.1 (3)	C9—C10—C11—C12	0.0 (3)
C8—N1—C7—C6	177.94 (15)	C9—C10—C11—C15	178.80 (19)
C5—C6—C7—O1	−152.85 (18)	C10—C11—C12—C13	0.7 (3)
C1—C6—C7—O1	27.0 (2)	C15—C11—C12—C13	−178.2 (2)
C5—C6—C7—N1	28.1 (2)	C9—N3—C13—C12	0.4 (4)
C1—C6—C7—N1	−152.03 (16)	C11—C12—C13—N3	−0.9 (4)

### Hydrogen-bond geometry (Å, º)

**Table:**

*D*—H···*A*	*D*—H	H···*A*	*D*···*A*	*D*—H···*A*
N2—H1*N*2···O1	0.82 (2)	1.94 (2)	2.644 (2)	144 (2)
N1—H1*N*1···S1^i^	0.81 (2)	2.74 (2)	3.5106 (15)	157.8 (18)
C10—H10*A*···S1	0.93	2.57	3.2211 (19)	127

Symmetry code: (i) −*x*+1, −*y*+2, −*z*+1.
